# Psychometric Properties of the Heteronormative Attitudes and Beliefs Scale: A Multigroup Analysis in a Greek Sample

**DOI:** 10.1007/s10508-026-03480-8

**Published:** 2026-06-01

**Authors:** Ioanna Fotopoulou, Konstantinos Christos Daoultzis, Panagiotis Kordoutis

**Affiliations:** Department of Psychology, Panteion University of Social and Political Sciences, 136, Syngrou Ave, 17671 Athens, Greece

**Keywords:** Heteronormative Attitudes and Beliefs Scale, Heteronormativity, Greek population, Mental health professionals, Heterosexism, Sexual orientation

## Abstract

Heteronormativity refers to a hierarchical system of beliefs, attitudes, and social practices grounded on the assumption that there are only two distinct genders, and that heterosexuality represents the only “normal,” desirable, and acceptable form of sexual behavior. Given the absence of a validated measure assessing heteronormativity in Greece, this study examined the reliability and validity of the Heteronormative Attitudes and Beliefs Scale (HABS; Habarth, 2015) in a Greek sample of 973 straight and not exclusively heterosexuals (Sample 1 [non-mental health professionals]: *n* = 605, Sample 2 [mental health professionals]: *n* = 368). Multigroup analyses indicated that, while the basic factorial structure of the scale was supported, full measurement invariance was not achieved: differences in factor loadings, intercepts, and residuals suggest that the HABS may not assess heteronormativity equivalently across groups. Positive correlations between heteronormativity, homonegativity, and negative attitudes towards lesbians and gay men, as well as negative correlation between heteronormativity and openness to experience supported the convergent and discriminant validity of the HABS. Contrary to expectations, only the Essential sex and Gender beliefs factor was positively associated with social desirability in Sample 1. Overall, the HABS appears to be a reliable and valid instrument for assessing heteronormativity and can be confidently employed in future psychological research in the Greek context.

## Introduction

Sexual minority and gender identities, such as gay, lesbian, bisexual, and transgender individuals, often face stigmatization and negative attitudes due to non-conformity with dichotomous social expectations regarding sexual orientation and gender identity (Habarth, 2015; Seal, 2019). These attitudes are rooted in heteronormativity, a belief system asserting that there are only two genders, aligned strictly with biological sex, and that heterosexuality is the default orientation (Butler, 1990; Kitzinger, 2005; Kowalski & Scheitle, 2020). Heteronormativity encompasses essentialist and binary views of gender, prescribing rigid roles and behaviors for men and women, and rejecting fluidity in gender identity and expression (Bogoslavsky, 2022; Duncan et al., 2019; Ferrari et al., 2021; Habarth, 2008, 2015; Rich, 2003; Seal, 2019; Warner, 1993).

Heteronormativity functions as a social hierarchy that privileges certain groups, such as men, cisgender identities, and heterosexual orientations, while marginalizing others, including women, intersex variations, and homosexual and bisexual orientations (Ferrari et al., 2021; Scandurra et al., 2021). It constructs men and women as sexually opposite but complementary in a manner that reinforces men’s superiority and hegemony (Knudson-Martin & Mahoney, 2009; Martino, 2000; Tolman, 2006). As it is embedded in cultural norms (Bible, 2020), heteronormativity affects many areas of life (Lahad, 2013). Through rules, policies, and informal interactions that normalize binary gender and attraction to the opposite sex (van der Toorn et al., 2020; Williams et al., 2009), it becomes established in organizations and social institutions such as education (Enson, 2015), workplace (Corlett et al., 2022), and health (Vergara, 2020).

Heterosexism, a concept closely related to heteronormativity, refers to the belief that heterosexuality is preferable and morally superior (Altman et al., 2012; Robertson, 2017). While heteronormativity establishes the boundaries of socially acceptable identities and relationships, heterosexism specifically targets non-heterosexual individuals (Habarth, 2015). Heteronormativity is considered a fundamental precursor to heterosexism (Habarth et al., 2019), encompassing negative attitudes, biases, behaviors, and even violence toward gender roles and sexual orientations that deviate from heterosexual norms, such as homosexuality and bisexuality (Bogoslavsky, 2022). Consequently, individuals with minority sexual identities are experiencing significantly high minority stress (Meyer, 2003; Ward & Schneider, 2009).

Research has shown that heteronormativity is positively associated with homonegativity, defined as negative attitudes towards sexual minorities which can lead to aversion and even physical or verbal violence against homosexual individuals (Habarth, 2015; Herek, 2004; Morrison & Morrison, 2003). Studies have found strong correlations between heteronormativity and factors such as gender and sexual orientation with heteronormativity being more prevalent among heterosexual cisgender men compared to women and individuals with minority sexual or gender identities (Cartwright et al., 2016; Habarth, 2015; Scandurra et al., 2021; Zsila et al., 2021). Research has also highlighted factors such as religiosity, personality traits like openness to experience, and social desirability are essential in understanding the core dynamics of heteronormativity.

Religiosity, defined as the degree to which individuals adhere to religious beliefs and participate in religious communities (Schaub et al., 2017), has been consistently associated with higher levels of heteronormativity and homonegativity, particularly in societies with strong Christian, Muslim, or Jewish traditions (Chapman et al., 2011; Elboim-Gabyzon et al., 2023; Georgiou & Fotiou, 2020; Herek & Capitanio, 1996; Herek & McLemore, 2013; Marsh & Brown, 2011; Scandurra et al., 2021). As Sakalli (2002) notes, since homosexuality is often considered sinful within the major monotheistic religions (Christianity, Islam, Judaism), more conservative and traditional religious individuals are more likely to hold negative attitudes towards homosexuality (Harbaugh & Lindsey, 2015).

Openness to experience, defined as a personality trait characterized by curiosity and interest in novel ideas and people (Ashton & Lee, 2009), has been consistently found to be negatively associated with heteronormativity and negative attitudes toward sexual minorities (Cullen et al., 2002; Habarth, 2008, 2015; Huffaker & Kwon, 2016; Scandurra et al., 2021; Sibley & Duckitt, 2008). Due to the perceived “unfamiliarity” of LGBTQI+ individuals and identities, those with lower levels of openness are more likely to hold negative attitudes, while individuals with higher openness tend to be less heteronormative.

Habarth (2008, 2015) found that social desirability is positively correlated with heteronormativity. Social desirability refers to the tendency of individuals to respond to survey questions in ways that present themselves favorably to others (Paulhus, 1991). Individuals with a higher need for social approval are more likely to endorse and conform to dichotomous views of gender, reinforcing heteronormative beliefs and behaviors.

### Heteronormativity in Greece

Greek society is characterized by traditional and conservative values, where heterosexuality is normalized and perceived as dominant, while non-heteronormative identities are viewed as “abnormal” and potentially dangerous (Grigoropoulos, 2022; Papanastasiou, 2020). Negative attitudes towards same-sex relationships and marriage are significantly predicted by religiosity as well gender, with women generally being more accepting than men (Grigoropoulos, 2019; Grigoropoulos & Kordoutis, 2015; Konstantinidis et al., 2021). The Greek Orthodox Church, a significant influence on societal attitudes, regards homosexuality as sinful, and non-heterosexual family structures are seen as harmful to the national well-being (Pettas et al., 2022).

Greek cultural norms have historically prioritized heterosexual marriage and heteronormative parenthood as central to family life (Grigoropoulos, 2022; Voultsos et al., 2019). Until recently, institutional barriers included the exclusion of same-sex couples from joint adoption. In February 2024, Greece enacted marriage equality (Law 5089/ 2024), extending full marital and parental recognition to same-sex couples, while leaving surrogacy unchanged -permitted only for women on medical grounds and therefore inaccessible to same-sex couples of men. Beyond formal law, subtler forms of discrimination, including workplace microaggressions, remain common and compel LGBTQI+ individuals to adopt coping strategies (Papadaki et al., 2021). Public attitudes also appear ambivalent: World Values Survey Wave 7 results place Greece at a mid-range level of acceptance of LGBTQI+ people (approximately 65%), comparatively higher than its immediate neighbours, many of which remain strongly homophobic (Haerpfer et al., 2022). However, recent Greek and EU-Greece studies document persistent avoidance of public displays of affection and notable rates of harassment: for example, the FRA’s LGBTI survey states that 33% of respondents in Greece experienced harassment in the past year and 13% an attack in the past five years because of their identity (European Union Agency for Fundamental Rights, 2020), while the majority of LGBTQI+ students are hiding their sexual identity at school (European Union Agency for Fundamental Rights, 2020).

Heteronormativity is also reflected in gender inequality, with Greece ranking last among European countries in gender equality (European Institute for Gender Equality, 2020). Women remain significantly underrepresented in government positions and experience disproportionate levels of gender-based violence. Domestic violence and femicides are prevalent, with women being four times more likely than men to be victims of domestic violence (Karakasi et al., 2022). These findings highlight the urgent need for research aimed at understanding the scope and impact of heteronormativity in Greek society, with a focus on promoting inclusivity and equality.

### Measurement of Heteronormativity: Heteronormative Attitudes and Beliefs Scale

The Heteronormative Attitudes and Beliefs Scale (HABS) by Habarth (2015) is the only available comprehensive tool for assessing heteronormativity in international literature. The HABS consists of 16 items across two factors: “Essential Sex and Gender,” which measures essentialist beliefs about gender as aligned with biological sex, and “Normative Behavior,” which assesses attitudes toward expected heterosexual behaviors within binary gender roles. The scale has been validated in various contexts, including Italy, a country with a cultural environment like Greece, where its psychometric properties were confirmed, although four items had loadings below .40. The study also demonstrated convergent and criterion validity through correlations with homonegativity, religiosity, and openness to experience (Scandurra et al., 2021). A shorter, eight-item version of the scale has also been validated in Chile (Orellana et al., 2022).

The HABS has been widely used in research on heteronormativity (Cartwright et al., 2016; Habarth et al., 2019; Johnson et al., 2021; Orellana et al., 2022; Rothblum et al., 2017; Schaub et al., 2017; Torres Rosado, 2019; Zsila et al., 2021). In Greece, while several scales exist to measure prejudice toward sexual and gender minorities, such as the Greek version of the Attitudes Toward Lesbians and Gay Men scale (Grigoropoulos et al., 2010), and the Beliefs about Children’s Adjustment in Same-Sex Families Scale (Daoultzis & Kordoutis, 2025), no tool specifically designed to comprehensively assess heteronormativity has been developed or validated. This gap underscores the need for a dedicated instrument to measure heteronormativity in the Greek context, given its significant social and institutional impact.

### The Current Study

Given the literature and practical gap in assessing heteronormativity in Greece, this study examined the psychometric properties of the Greek version of the HABS. The psychometric accuracy of the scale was tested in two distinct samples: non-mental health professionals (Sample 1) and mental health professionals, including psychologists, psychotherapists, counselors, psychiatrists, social workers in mental health contexts, and psychology students (Sample 2).

The inclusion of mental health professionals was deemed necessary for several reasons. First, there is a lack of data on heteronormativity levels among mental health professionals in Greece, and no reliable tool exists for measuring it in this population. Previous research has focused on other professional groups, such as social workers and students (Papadaki & Papadaki, 2011; Papadaki et al., 2013; Schaub et al., 2017), but not on mental health professionals, particularly psychologists. Additionally, the attitudes of mental health professionals toward their clients’ sexual orientation can significantly influence the services they provide (Biaggio et al., 2000; Thompson et al., 2015). As mental health professionals are responsible for addressing the consequences of minority stress and its psychological effects, it is essential for them to develop the skills necessary to work effectively with LGBTQI + individuals (Ginicola et al., 2017). The American Psychological Association has emphasized the ethical mandate to eliminate heterosexist biases in practice with these populations (APA, 2021, as cited in Shin et al., 2023).

The study aimed to (1) confirm the two-factor structure of the Greek version of the HABS in both non-mental health professionals and professionals, (2) examine configural, metric, and scalar invariance across the two samples, (3) provide evidence of convergent and discriminant validity by exploring correlations between heteronormativity and other related concepts, and (4) assess the internal reliability of the scale and its subscales.

## Method

### Participants

A total of 973 individuals participated in the study (Sample 1 or non-mental health professionals: *n* = 605, Sample 2: *n* = 368), with a mean age of 29.31 years (SD = 10.94, range, 18–69) and 30.35 years (SD = 7.40, range, 18–65), respectively. Specifically, in Sample 2 (mental health professionals), there were 251 (68.2%) psychologists, 33 (8.9%) psychiatrists, and social workers working in the field of mental health, 32 (8.7%) psychotherapists, and counselors, 29 (7.9%) unemployed individuals who had previously worked in such areas, and 23 (6.3%) undergraduate psychology students.

### Measures

#### Translation, Back-Translation

The items from all scales were initially translated and culturally adapted from English to Greek by three independent bilingual researchers (forward translation). Minor modifications were made to ensure maximum equivalence between the translated and original items. Subsequently, the items were back translated into English, and the final version was reviewed by a professional translation editor specializing in social psychology. This multi-step approach follows best practices recommended in the literature (Brislin, 1970; Cha et al., 2007; Gudmundsson, 2009; Swami & Barron, 2019).

##### Kinsey Scale

The Kinsey Scale (Kinsey et al., 1948) was used to assess the sexual orientation of the participants. The scale consists of seven points, and participants could respond from 1 (Exclusively heterosexual) to 7 (Exclusively homosexual). For the purposes of the present study, responses of 1 were recoded as “straight,” while responses of 2–7 were grouped as “not exclusively heterosexual.” This dichotomization is consistent with prior research adopting the minority stress perspective (e.g., Meyer, 2003) and large-scale surveys where sexual minority participants are combined into a single category for statistical analysis. However, it should be noted that this coding collapses intermediate positions on the Kinsey scale and does not differentiate bisexual or “mostly heterosexual” identities (see also Savin-Williams & Vrangalova, 2013).

##### Religiosity

Following the approach of previous studies (e.g., Georgiou & Fotiou, 2020; Lingiardi et al., 2016; Scandurra et al., 2021), religiosity was assessed using two researcher-developed items The first item measured the frequency of religious practices (“How often do you perform your religious duties?”) on a scale from 1 (Never) to 7 (Every day). The second item assessed personal engagement with religious or spiritual issues (“How much do you generally concern yourself with religious-spiritual issues?”) on a scale from 1 (Not at all) to 7 (Very much). The two religiosity items were analyzed separately rather than combined into a single index, as the inter-correlation between the two religiosity items was modest in both samples (Sample 1: *r* = .49, *p* < .001; Sample 2: *r* = .43, *p* < .001). The Spearman–Brown coefficients were .66 and .61 respectively, which both fall below the acceptable threshold (Eisinga et al., 2013).

##### Heteronormative Attitudes and Beliefs Scale (HABS)

Heteronormativity was measured using the Greek version of the HABS (Habarth, 2015), a seven-point Likert scale (1 = Strongly disagree, 7 = Strongly agree). The scale consists of 16 items, divided into two factors: Essential Sex and Gender, which reflects beliefs about the binary nature of gender (e.g., “All people are either male or female”), and Normative Behavior, which captures attitudes toward traditional heterosexual roles (e.g., “The best way to raise a child is to have a mother and a father raise the child together”). Eight items are reverse-coded. Subscale scores were computed as the mean of the corresponding items. Higher scores on each subscale indicate stronger heteronormative beliefs. In the present study, internal consistency for the Essential Sex and Gender subscale was α = .87 in Sample 1 and α = .79 in Sample 2. For Normative Behavior, α = .89 in Sample 1 and α = .76 in Sample 2. Full results regarding the reliability and validity of the scale are presented in the relevant sections of the Results.

##### Modern Homonegativity Scale (MHS)

The MHS (Morrison & Morrison, 2003) was used to assess contemporary negative attitudes toward gay men and lesbian women. Unlike other measures of homonegativity, the MHS does not focus on traditional, moral, or religious objections. It is a single-factor tool with two parallel 12-item versions: one for gay men (e.g., “Gay men should stop shoving their lifestyle down other people’s throats”) and one for lesbian women (e.g., “In today’s tough economic times, tax dollars shouldn’t be used to support lesbian organizations”). Responses are rated on a five-point Likert scale (1 = Strongly agree, 5 = Strongly disagree). Both versions have demonstrated strong reliability (α > .90) and construct validity (e.g., positive correlations with modern racism and sexism). In this study, reliability coefficients for the gay men and lesbian women versions were α = .94 and α = .95 (McDonald’s ω = .94 and McDonald’s ω = .95) in Sample 1, and α = .91 and α = .93 (McDonald’s ω = .92 and McDonald’s ω = .95) in Sample 2, respectively. Higher scores indicate greater homonegativity, with two items requiring reverse coding.

##### Attitudes Toward Lesbians and Gay Men (ATLG; Herek, 1994)

The Greek version of ATLG scale (Grigoropoulos et al., 2010) was used in this study. The scale comprises 20 items (e.g., “I would not be too upset if I learned that my son was a homosexual”) rated on a seven-point Likert scale (1 = Strongly disagree, 7 = Strongly agree). Total scores were calculated by summing item responses, with certain items reverse-coded. Higher scores reflect more negative attitudes toward gay and lesbian individuals. The scale’s psychometric properties have been confirmed, with internal consistency reliability of α = .91 and test–retest reliability of r = .91 (Grigoropoulos et al., 2010). In this study, internal consistency was α = .95 (McDonald’s ω = .96) for Sample 1 and α = .90 (McDonald’s ω = .91) for Sample 2.

##### Openness to Experience Subscale

Openness to Experience was assessed using the subscale from the HEXACO-60 questionnaire (Ashton & Lee, 2009). This subscale consists of 10 items (e.g., “People have often told me that I have a good imagination”) rated on a five-point Likert scale (1 = Strongly disagree, 5 = Strongly agree). Higher scores reflect greater openness to experience, with five items requiring reverse coding. Previous studies have reported internal consistency reliability of α = .77 in student samples and α = .80 in community samples. In the present study, internal consistency was α = .75 (McDonald’s ω = .75) for Sample 1 and α = .74 (McDonald’s ω = .73) for Sample 2.

##### Personal Reaction Inventory

The Personal Reaction Inventory (Reynolds, 1982) is a shorter alternative to the Marlowe-Crowne Social Desirability Scale (SDS, 1961) with acceptable construct validity and psychometric properties. It contains 13 items (e.g., “I am always willing to admit it when I make a mistake”) compared to the 33 items in the original SDS. Items are answered in a True–False format, with most items reverse-coded. Higher scores indicate greater social desirability. The scale demonstrated acceptable internal consistency by using the KR-20 index (for Sample 1: KR-20 = .71 and Sample 2: KR-20 = .78).^1^

##### Demographic Questions

In addition to the items included in the established scales, participants also answered questions assessing demographic characteristics. These included age, gender, education level, profession, place of residence, and relationship status. Furthermore, participants were asked whether they had personal contacts with LGBTQI+ individuals (“Do you have contacts with LGBTQI + individuals, e.g., lesbians, gay men, bi people, trans or intersex people?”, response options: 1 = Yes, 2 = No).

### Procedure

Sampling for the study was conducted online in June and July 2024. Online data collection was chosen for its immediacy and its potential to reduce socially desirable responses compared to face- to-face methods, particularly in studies involving sensitive questions (Pealer et al., 2001; Turner et al., 1998). Two identical questionnaires were created using Google Forms and distributed electronically. Participants were informed about the study’s purpose, the anonymous and voluntary nature of participation, and provided written consent. They then completed demographic information, two religiosity questions, the Greek version of the HABS, and four other scales. The average completion time was 15 min. No compensation was provided. For Sample 1, a non-probabilistic online snowball sampling method was used (Baltar & Brunet, 2012). The questionnaire link was shared with the authors’ personal contacts, on social media platforms, and via email to university secretariats, encouraging students to participate and share the link within their networks. For Sample 2, purposive non-probabilistic sampling was employed. The questionnaire link was disseminated through a Greek online psychology magazine, the authors’ LinkedIn profiles, personal messages, and emails to mental health professionals listed on official websites.

### Analytic Strategy

The data were analyzed using IBM SPSS, version 29. Differences between the two samples were tested using independent samples *t*-tests and chi-square tests of independence. The reliability of the Greek version of the HABS was assessed using Cronbach's alpha coefficient. Pearson correlation analysis was used for bivariate correlations and, consequently, to test the convergent and discriminant validity of the scale. For comparing the internal structure of the tool, the multigroup confirmatory factor analysis was employed. A baseline model was initially created for Sample 1 and Sample 2 separately, without imposing constraints on parameters (Tabachnick & Fidell, 2019), in AMOS, Version 26. Following assessment of fit of the baseline configural model (configural invariance), a series of constrained models was generated in which equality constraints were imposed upon error covariances, factor loadings (metric invariance), and item intercepts (scalar invariance). Metric invariance and scalar invariance were tested in separate nested models; thus, for comparison of nested models, equality constraints of previous models (e.g., factor loadings) were maintained while additional constraints (e.g., item intercepts) were added to subsequent models. The significance level alpha was set at .05 (5% error—95% confidence interval) for all analyses.

## Results

### Differences Between the Two Samples

Between Sample 1 and Sample 2, differences were found in participants’ gender (*χ*^2^ = 6.34, *p* = .01, Cramer’s V = .08), education (*χ*^2^ = 5.12, *p* = .03, Cramer’s V = .07), current place of residence (*χ*^2^ = 6.92, *p* = .01, Cramer’s V = .08), relationship status (*χ*^2^ = 12.59, *p* < .001, Cramer’s V = .11), contact or no contact with LGBTQI+ individuals (*χ*^2^ = 16.29, *p* < .001, Cramer’s V = .13), and frequency of performing religious duties (*t* = 2.63, *p* = .004, *d* = .17). No differences were found in age (*t* = -1.63, *p* = .10, *d* = -.11), sexual orientation (*χ*^2^ = .98, *p* = .75, Cramer’s V = .01), and level of personal engagement with religious or spiritual issues (*t* = 2.63, *p* = .17, *d* = .09). Regarding the key variables of the study, significant differences emerged in HABS (*t* = 6.19, *p* < .001, *d* = .41), MHS (*t* = 7.98,* p* < .001, *d* = .53), ATLG (*t* = 6.65, *p* < .001, *d* = .44), and Social Desirability (*t* = 3.27, *p* = .001, *d* = .22), while no difference was observed in Openness to Experience (*t* = -0.31, *p* = .38, *d* = -.02). Table 1 presents detailed demographic characteristics and differences between the two samples.

**Table 1:** Demographic characteristics

	Non-mental health professionals (Sample 1) (*n* = 605)	Mental health professionals (Sample 2) (*n* = 368)	*t/χ*^2^	*p-*value	Cohen’s *d /* Cramer’s *V*
Age, in years	29.31 ± 10.94	30.35 ± 7.40	–1.63	0.10	–0.11
*Gender*			6.34	0.01	0.08
Men	165 (27.3)	74 (20.1)			
Women	440 (72.7)	294 (79.9)			
*Sexual Orientation*			0.98	0.75	0.01
Straight	376 (62.1)	225 (61.1)			
Not exclusively heterosexual	229 (37.9)	143 (38.9)			
*Education*			5.12	0.03	0.07
Up to high school	216 (35.7)				
Undergraduate	217 (35.9)	146 (39.7)			
Master/Ph.D	172 (28.4)	222 (60.3)			
*Profession*					
University students	294 (48.6)	23 (6.3)			
Employed	279 (46.2)	316 (85.8)			
Unemployed	32 (5.2)	29 (7.9)			
*Residence*			6.92	0.01	0.08
Major cities	278 (46)	212 (57.6)			
Rural areas	245 (54)	156 (42.3)			
*Relationship status*			12.59	< 0.001	0.11
Single	241 (39.8)	109 (29.6)			
Partnered	364 (60.2)	259 (70.4)			
*Interactions with* *LGBTQI* + *individuals*			16.29	< 0.001	0.13
Yes	464 (76.7)	321 (87.2)			
No	141 (23.3)	47 (12.8)			
*Religiosity*					
Frequency of religious practices	1.15 ± 0.36	1.09 ± 0.29	2.63	0.004	0.33
Personal engagement	3.26 ± 1.93	3.09 ± 1.78	1.37	0.17	0.09
*HABS*	2.99 ± 1.30	2.52 ± 0.83	6.19	< 0.001	0.41
*MHS*	59.81 ± 26.31	47.24 ± 19.02	7.98	< 0.001	0.53
*ATLG*	39.12 ± 24.83	29.85 ± 12.65	6.65	< 0.001	0.44
*Openness*	4.06 ± 0.61	4.07 ± 0.55	–0.31	0.38	–0.02
*Social desirability*	18.53 ± 2.15	18.07 ± 2.21	3.27	0.001	0.22

HABS = Heteronormative Attitudes and Beliefs Scale; MHS = Modern Homonegativity Scale; ATLG = Attitudes Toward Lesbians and Gay Men; PRI = Personal Reaction Inventory. Values for HABS, MHS, ATLG, Openness, and PRI are presented as means (SDs). Group comparisons were conducted using *t*-tests for continuous variables and *χ*^2^ tests for categorical variables. Effect sizes are reported as Cohen’s *d* for continuous variables and Cramer’s V for categorical variables. Additional descriptives: HABS (Range = 6, Min = 1, Max = 7, Variance = 1.36); MHS (Range = 96, Min = 24, Max = 120, Variance = 603.93); ATLG (Range = 120, Min = 20, Max = 140, Variance = 463.76); Openness (Range = 3.80, Min = 1.20, Max = 5, Variance = 0.35); PRI (Range = 11, Min = 13, Max = 24, Variance = 4.75)

### Validity

#### Multigroup Analysis

**Fig. 1:**
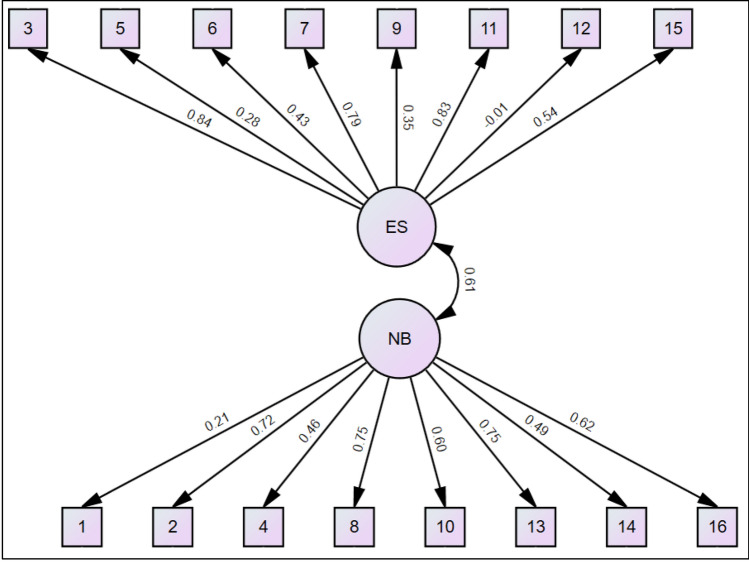
Two-factor model for non-mental health professionals (Sample 1). *Note:* The loadings are standardized. ES = Essential Sex and Gender, NB = Normative Behavior

**Fig. 2:**
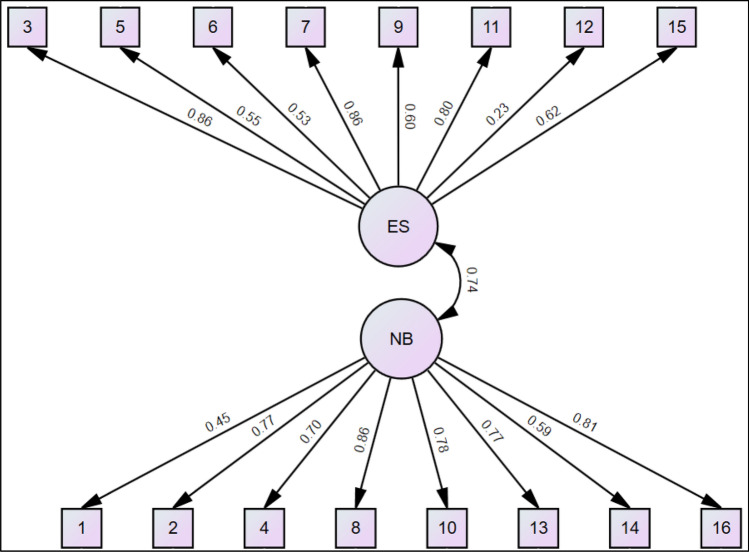
Two-factor model for mental health professionals (Sample 2). *Note:* The loadings are standardized. ES = Essential Sex and Gender, NB = Normative Behavior

A multigroup structural equation modeling was performed to test for structural differences between the two groups. The analysis tested for configural, metric, and scalar invariance across the groups. The following models were compared: the unconstrained model (configural invariance), the constrained model for factor loadings (metric invariance), the model with constrained intercepts (scalar invariance), and the model with constrained error variances (see also Table 2 and Figs. 1 and 2).

**Table 2:** Model fit indices for multigroup confirmatory factor analysis

**Model**	*χ*^2^ *(df)*	*RMSEA*	*CFI*	*TLI*	*Δχ*^2^ *(df)*	*p-*value
Configural Invariance	1050.90 (206)	0.06	0.88	0.86	-	-
Metric Invariance (Loadings)	1170.02 (220)	0.06	0.86	0.85	119.11 (14)	< 0.001
Scalar Invariance (Intercepts)	1272.19 (223)	0.07	0.85	0.84	102.17 (3)	< 0.001
Strict Invariance (Residuals)	1472.07 (239)	0.07	0.82	0.82	302.05 (19)	< 0.001

RMSEA = Root Mean Square Error of Approximation; CFI = Comparative Fit Index; TLI = Tucker-Lewis Index. Significant *χ*^2^ difference tests (*Δχ*^2^) indicate variance at each level (loadings, intercepts, residuals).

The baseline fit of the unconstrained model (configural invariance) showed adequate fit to the data: *χ*^2^(206) = 1050.91, *p* < .001, RMSEA = .065, CFI = .910, TLI = .930. This indicates that the two-factor structure of the HABS (essentialist beliefs and normative behaviors) fits both groups well without any equality constraints. When factor loadings were constrained to be equal across the two groups (metric invariance), the fit of the model worsened slightly, *χ*^2^(220) = 1170.03, *p* < .001, RMSEA = .067, CFI = .865, TLI = .853. The significant difference in chi-square values between the configural and metric models [*Δχ*^2^(14) = 119.12, *p* < .001] suggests that the factor loadings were not equivalent across the two samples. Next, we tested scalar invariance by constraining the intercepts across groups. The scalar invariance model showed further deterioration in fit, *χ*^2^(223) = 1272.20, *p* < .001, RMSEA = .070, CFI = .851, TLI = .840, and the chi-square difference was significant [*Δχ*^2^(3) = 102.17, *p* < .001], indicating that intercepts were also not equivalent between the two groups. Finally, constraining the residuals (strict invariance) led to an even poorer fit, *χ*^2^(239) = 1472.07, *p* < .001, RMSEA = .073, CFI = .825, TLI = .824. The significant chi-square difference [*Δχ*^2^*(*19) = 302.05, *p* < .001] suggests that error variances were also not invariant across groups.

The nested model comparisons confirmed that measurement weights, structural covariances, and residuals all differed significantly between the two groups. For instance, the chi- square difference between the configural and metric models was significant [*Δχ*^2^(14) = 119.19, *p* < .001], and similarly, the difference between the metric and scalar models was also significant [*Δχ*^2^(3) = 102.17, *p* < .001]. The chi-square difference between the scalar and strict invariance models [*Δχ*^2^(19) = 302.05, *p* < .001] further indicates non-invariance in the error variances.

Overall, the multigroup analysis indicated that while the basic two-factor structure of the HABS was consistent across the two samples (configural invariance), there were significant differences in factor loadings, intercepts, and residuals between the groups. This suggests that the two groups may conceptualize and respond to the items on the HABS differently. These findings imply that further refinement of the scale may be needed for it to function equivalently across different populations. See also Table 3 for the factor loadings, intercepts, and residuals which were not invariant across groups.

**Table 3:** Invariant-variant factor loadings, intercepts, and residuals in multigroup analysis

**Item number**	Factor Loadings (Metric Invariance)	Intercepts (Scalar Invariance)	Residuals (Strict Invariance)
*Essential Sex and Gender*			
3. There are only two sexes: male and female	Invariant	**Variant**	Invariant
5. Gender is the same thing as sex	**Variant**	Invariant	Invariant
6. Femininity and masculinity are determined by biological factors, such as genes and hormones, before birth	**Variant**	**Variant**	Invariant
7. All people are either male or female	Invariant	**Variant**	**Variant**
9. Gender is a complicated issue, and it doesn’t always match up with biological sex. (R)	**Variant**	Invariant	**Variant**
11. People who say that there are only two legitimate genders are mistaken. (R)	Invariant	**Variant**	**Variant**
12. Gender is something we learn from society. (R)	Invariant	**Variant**	Invariant
15. Sex is complex; in fact, there might even be more than two sexes. (R)	**Variant**	Invariant	Invariant
*Normative Behavior*			
1. In healthy intimate relationships, women may sometimes take on stereotypical “male” roles, and men may sometimes take on stereotypical “female” roles. (R)	**Variant**	Invariant	Invariant
2. In intimate relationships, women and men take on roles according to gender for a reason; it’s really the best way to have a successful relationship	Invariant	**Variant**	**Variant**
4. People should partner with whomever they choose, regardless of sex or gender. (R)	**Variant**	**Variant**	Invariant
8. Things go better in intimate relationships if people act according to what is traditionally expected of their gender	Invariant	**Variant**	Invariant
10. It’s perfectly okay for people to have intimate relationships with people of the same sex. (R)	**Variant**	**Variant**	**Variant**
13. There are particular ways that men should act and particular ways that women should act in relationships	**Variant**	Invariant	Invariant
14. The best way to raise a child is to have a mother and a father raise the child together	Invariant	Invariant	**Variant**
16. Women and men need not fall into stereotypical gender roles when in an intimate relationship. (R)	Invariant	**Variant**	Invariant

Items highlighted as “Variant” indicate statistically significant differences in factor loadings, intercepts, or residuals between the non-mental health professionals and mental health professionals. Factor loadings (Metric invariance): Variant factor loadings indicate differences in the strength of the relationships between latent variables and their indicators across groups. Intercepts (Scalar invariance): Variant intercepts suggest that the groups differ in their mean levels of the observed variables. Residuals (Strict invariance): Variant residuals indicate differences in measurement error between groups. Items marked with (R) are reverse-coded

#### Convergent and Discriminant Validity

To assess the convergent validity of the HABS, its two factors (Essential Sex and gender and Normative Behavior) were examined for their correlations with Homonegativity toward Gay Men, Homonegativity toward Lesbians, Negative Attitudes toward Lesbians and Gay Men, Social desirability and the two items of religiosity (frequency of performing religious duties and personal engagement with religious / spiritual matters). To test the discriminant validity of the HABS, Openness to Experience was used. Table 4 presents the results of the correlation analyses in Sample 1 and Sample 2.

**Table 4:** Correlations of the HABS factors with the other variables for the non-mental (Sample 1) and mental health professionals (Sample 2)

	1	2	3	4	5	6	7	8	9
1. ES	–	0.70***	0.73***	0.73***	0.68***	–0.25**	0.09*	0.33***	0.28***
2. NB	0.55***	–	0.78***	0.78***	0.84***	–0.22**	0.08	0.37***	0.33***
3. Homonegativity towards gay	0.61***	0.64***	–	0.97***	0.75***	–0.24**	0.04	0.32***	0.31***
4. Homonegativity towards lesbians	0.60***	0.63***	0.96***	–	0.74***	–0.25**	0.03	0.32***	0.28***
5. ATLG	0.52***	0.69***	0.68***	0.67***	–	–0.18**	0.09*	0.41***	0.36***
6. Openness to experience	–0.15**	–0.18**	–0.21**	–0.21**	–0.19**	–	–0.12**	–0.06	0.07
7. Social desirability	0.05	0.03	0.04	0.05	0.07	–0.17**	–	–0.08	–0.03
8. Religious duties	0.22***	0.24***	0.21***	0.19***	0.29***	0.01	–0.10	–	0.49***
9. Engagement with religion	0.24***	0.25***	0.24***	0.23***	0.23***	0.10	0.01	0.44***	–

Correlations for the non–mental health professionals are presented below the diagonal, and correlations for the mental health professionals are presented above the diagonal

ES = Essential Sex and Gender, NB = Normative Behavior, ATLG = Attitudes Toward Lesbians and Gay Men

^*^p < 0.05, **p < 0.01, ***p < 0.001

Correlation analyses provided evidence for both convergent and discriminant validity of the HABS. Regarding convergent validity, both HABS factors (Essential Sex and Gender or ES, Normative Behavior or NB) were strongly and positively correlated with Homonegativity toward gay men and toward lesbians (Sample 1: for ES *r* = .73, for NB *r* = .78, all *p* < .001; Sample 2: for ES *r* = .61, for NB *r* = .64, all *p* < .001), as well as with Negative Attitudes Toward Lesbians and Gay Men (ATLG; Sample 1: for ES *r* = .68, for NB *r* = .84, all *p* < .001; Sample 2: for ES *r* = .52, for NB *r* = .69, all *p* < .001). In addition, both factors showed significant positive associations with religiosity, including frequency of religious practices (Sample 1: for ES *r* = .33, for NB *r* = .37; Sample 2: for ES *r* = 0.22, for NB *r* = .24; all *p* < 0.001) and personal engagement with religion (Sample 1: for ES *r* = 0.28, for NB *r* = 0.33; Sample 2: for ES *r* = 0.24, for NB *r* = 0.25; all *p* < 0.001). In terms of discriminant validity, the two HABS factors were only modestly negatively correlated with Openness to Experience (Sample 1: ES,* r* = −0.25, *p* = 0.004; NB, *r* = −0.22, *p* = 0.007; Sample 2: ES, *r* = −0.15, *p* = 0.016; NB, *r* = −0.18, *p* = 0.009). Their associations with Social Desirability were negligible and nonsignificant (Sample 1: ES, *r* = −0.09, *p* = .063; NB *r* = −0.08, *p* = 0.078; Sample 2: ES *r* = 0.05, *p* = 0.326; NB, *r* = 0.03, *p* = 0.547). Finally, the inter-factor correlations (Sample 1: *r* = 0.70; Sample 2: *r* = 0.55, all *p* < 0.001) indicated related but distinct constructs, further supporting discriminant validity.

Fisher’s *z* tests were conducted to compare the magnitude of correlations across groups. Significant differences were observed for several correlations, including Essential Sex and Gender – Normative behavior (*z* = 3.27, *p* = 0.001), Essential Sex and Gender – Homonegativity toward gay men (*z* = 2.84, *p* = 0.004), Essential Sex and Gender – Homonegativity toward lesbians (*z* = 2.88, *p* = 0.004), Essential Sex and Gender – Attitudes towards lesbian and gay (*z* = 3.43, *p* = 0.001), Essential Sex and Gender – Social Desirability (*z* = –2.76, *p* = .006), Essential Sex and Gender – Religious duties (*z* = 2.09, *p* = .037), Normative behavior – Attitudes Towards Lesbian and Gay Men (*z* = 4.25, *p* < 0.001), Homonegativity toward gay men – Attitudes Towards Lesbian and Gay Men (*z* = 2.07, *p* = 0.039), Homonegativity toward lesbians – Attitudes Towards Lesbian and Gay Men (*z* = 2.03, *p* = 0.042), Attitudes Towards Lesbian and Gay Men – Religious duties (*z* = 2.48, *p* = 0.013), and Attitudes Towards Lesbian and Gay Men – Personal engagement with religion (*z* = 2.74, *p* = 0.006). All other differences were non-significant (*p* > 0.05).

### Reliability Analysis

Table 5 presents the internal consistency analyses for the two factors of the Greek version of the HABS across the two samples. In Sample 1 (non-mental health professionals), both factors demonstrated satisfactory internal consistency (Essential Sex and Gender: α = 0.87, ω = 0.86; Normative Behavior: α = 0.89, ω = 0.89). In Sample 2 (mental health professionals), reliability was somewhat lower but remained acceptable (Essential Sex and Gender: α = 0.79, ω = 0.82; Normative Behavior: α = 0.76, ω = 0.76). At the total-sample level, Cronbach’s α and McDonald’s ω were highly similar and above the recommended thresholds (Essential Sex and Gender: α = .81, ω = .83; Normative Behavior: α = .87, ω = .87). These results confirm that the Greek HABS demonstrates adequate internal consistency reliability, in line with the recommended cut-off points (α/ω ≥ .70 as acceptable and α/ω ≥ .80 as good; Kyriazos, 2017, p. 134).

**Table 5:** Cronbach’s alpha coefficients, correlations, and alpha if item deleted for the items of the HABS

	Cronbach’s alpha	McDonalds’ omega	Corrected Item- total correlation	Cronbach’s alpha if item deleted	McDonalds’ omega if item deleted
*Essential Sex and Gender total (8 items)*	0.81	0.83	0.35 to 0.75	0.75 to 0.85	0.77 to 0.87
Non-mental health professionals	0.87	0.86	0.51 to 0.78	0.82 to 0.87	0.82 to 0.87
Mental Health Professionals	0.79	0.82	0.36 to 0.72	0.71 to 0.80	0.75 to 0.83
*Normative Behavior total (8 items)*	0.87	0.87	0.36 to 0.77	0.84 to 0.88	0.83 to 0.88
Non-mental health professionals	0.89	0.89	0.41 to 0.81	0.86 to 0.90	0.87 to 0.90
Mental Health Professionals	0.76	0.76	0.22 to 0.63	0.71 to 0.79	0.70 to 0.79

“Corrected item–total correlation” reflects the correlation of each item with the sum of the remaining items. “Cronbach’s α if item deleted” and “McDonald’s ω if item deleted” indicate the reliability coefficient that would result if each item were removed individually; reported values represent the observed range across all items in the respective subscale, not cumulative deletions

## Discussion

Heteronormativity refers to a system of beliefs, attitudes, and social practices that position heterosexuality as the only “normal,” desirable, and acceptable form of sexual behavior (Montgomery & Stewart, 2012; Seal, 2019). This framework is a key precursor to various forms of prejudice and discrimination. Greek society is widely recognized as heteronormative (Papanastasiou, 2020; Pettas et al., 2022), where individuals with minority sexual and gender identities, including women and LGBTQI + individuals, continue to experience discrimination on multiple levels. To date, no tool has been validated for assessing heteronormativity in Greece. Thus, the aim of this study was to evaluate the psychometric properties of the HABS–the only scale in the international literature that exclusively and comprehensively measures heteronormativity–in a Greek sample comprising both non-mental health professionals and professionals. The latter group plays a critical role in providing mental health services to individuals with minority gender and sexual identities.

The findings indicate that the mental health professionals scored lower than the non–mental health group on all measures assessing heteronormativity and negative beliefs (HABS: *M* = 2.99 vs. *M* = 2.52; MHS: *M* = 59.81 vs. *M* = 47.24; ATLG: *M* = 39.12 vs. *M* = 29.85, respectively). Regarding the HABS structure, the final two-factor model demonstrated a good fit to the data in both populations. Nonetheless, the multigroup analysis also revealed several instances of variance between the two samples in the factor loadings, intercepts, and residuals. These findings suggest differences in how these two groups conceptualize and respond to items on the scale, which have implications for the validity of the HABS when applied across diverse populations.

The factor loadings for half the items (1, 5, 6, 9, 10, 13, and 15) were found to be variant. This indicates that the strength of the relationships between these items and the underlying latent factors (Essentialist Beliefs and Normative Behaviors) differs between the two samples. Such differences in factor loadings are critical because they imply that the two groups may not interpret the meaning of these items similarly (Byrne, 2016). For example, an item like “All people are either male or female” may resonate differently for mental health professionals, who are likely trained in more gender-inclusive frameworks, compared to non-mental health professionals, who may be more influenced by traditional gender norms. This variance of factor loadings suggests that the HABS may not assess heteronormativity equivalently across groups, making comparisons of factor means potentially problematic (Vandenberg & Lance, 2000).

Several items (2, 3, 6, 7, 10, 11, 12, 16) were variant in terms of intercepts. Variant intercepts indicate that the mean levels of the observed variables (i.e., item responses) differ between groups even when controlling for the latent variables. In other words, individuals from different groups may systematically respond differently to the same item, independent of their position on the underlying factor (Putnick & Bornstein, 2016). For example, the item “The best way to raise a child is to have a mother and a father raise the child together” might reflect deeply ingrained societal norms in the “general population” but could be seen as less relevant or appropriate among mental health professionals who are likely exposed to diverse family structures in their practice. Variance of intercepts is particularly concerning for group comparisons, as it suggests that observed differences may reflect measurement bias rather than true differences in heteronormative attitudes (Chen et al., 2005).

Lastly, variance was found also in several items’ residuals. This suggests differences in measurement error across groups, meaning that the reliability of these items varies between the mental health professionals and non-professionals. While strict invariance (including residuals) is not always required for meaningful comparisons across groups, significant differences in residuals can indicate that some items may be interpreted more ambiguously by one group compared to the other (Little, 2013). Variant residuals can affect the overall reliability and validity of the scale in a particular group (Byrne et al., 1989). These findings align with prior research that highlights the complexity of measuring heteronormativity across different populations. According to Barker (2014) and Jackson (2006), heteronormativity involves not only assumptions about sexuality but also intersects with gender, race, socioeconomic status, and religion, which influence how these norms are internalized and experienced differently in diverse groups.

Although the overall internal structure of the HABS is applicable to both populations, item 12 presents an issue specifically among mental health professionals. The factor loadings for the other items exceeded the minimum threshold of 0.30, and the absolute and incremental fit indices fell within acceptable ranges, indicating a good fit for the model. Previous research in countries such as Italy and Chile has similarly validated the conceptual structure of the HABS, with a reduced number of items for each factor. The Greek version appears to necessitate all 16 items proposed by the original scale, except for item 12, which displayed a loading below the acceptable limit (.30, as per Costello & Osborne, 2005, and 0.32, according to Tabachnick & Fidell, 2019). This lower loading might be attributed to translation challenges, as the Greek language lacks a direct equivalent for the word “gender.” Instead, a more descriptive phrase must be used, which may have led to reduced clarity or a shift in meaning compared to the original English version, “Gender is something we learn from society”. Future research should further investigate the performance of item 12 in Greek populations and consider excluding it from statistical analyses if its loading remains inadequate.

The convergent validity of the Heteronormativity scale was supported by positive correlations between its two factors and Homonegativity as well as Negative Attitudes towards Gay and Lesbian individuals, consistent with expectations. This correlation aligns with the notion that heteronormative beliefs are rooted in an ideological framework that promotes heterosexual relationships as the only acceptable form, while marginalizing non-heterosexual orientations and non-binary gender identities (Morrison & Morrison, 2003; Savin-Williams et al., 2010). In Sample 1, only the Essential Sex and Gender factor showed a significant positive correlation with Social desirability, which is consistent with previous findings (Habarth, 2008, 2015). However, neither the Normative Behavior factor in Sample 1 nor either factor in Sample 2 correlated significantly with social desirability. These results suggest that, within the Greek context, the tendency to respond in socially desirable ways is not strongly associated with beliefs about the gender binary or perceptions of heterosexual “normality,” diverging from earlier findings by Habarth (2008, 2015). It should be noted that although some correlations reached statistical significance due to the large sample size, their effect sizes were minimal (e.g., the correlation between Essential Sex and Gender beliefs and Social Desirability: *r* = 0.09; see also Table 4), accounting for less than 1% of the shared variance and thus negligible in practical terms.

The results also supported the convergent validity of the HABS in relation to religiosity. Specifically, both religious duties and engagement with religion were positively associated with essentialist beliefs about sex and gender, normative behavior expectations, and homonegative attitudes, across both samples. These findings are consistent with previous research showing that higher religiosity is linked to more traditional gender role attitudes and lower acceptance of sexual diversity (e.g., Adamczyk & Pitt, 2009; Jäckle & Wenzelburger, 2015). The consistent, moderate positive correlations suggest that the HABS captures value orientations theoretically aligned with religious worldviews emphasizing normativity and tradition. At the same time, the magnitude of these associations (*r* ≈ 0.20–0.40) indicate that the HABS does not merely reflect religious conservatism but rather taps into broader ideological orientations that resonate with, yet extend beyond, religiosity. In the Greek context, where Orthodox Christianity profoundly shapes cultural norms and public discourse on gender and sexuality (Grigoropoulos, 2019; Pettas et al., 2022), these results suggest that the HABS effectively captures the intersection between religious worldviews and heteronormative attitudes without conflating the two.

Discriminant validity was evidenced by the expected negative correlations between the HABS factors and Openness to Experience. A lack of this personality trait may foster discomfort with non-conventional ideas, including non-heterosexual orientations, as LGBTQI+ individuals, can represent unfamiliar or non-majority realities (Cullen et al., 2002; Habarth, 2008; Huffaker & Kwon, 2016; Scandurra et al., 2021; Sibley & Duckitt, 2008). In Greece, a “don’t ask, don’t tell” attitude toward homosexuality is prevalent, mirroring that observed in Italy, where tolerance of LGBTQI+ individuals is conditional on their invisibility in public or political spheres (Lingiardi et al., 2016). This invisibility likely reinforces perceptions of LGBTQI+ individuals as “foreign” or “unusual,” particularly among heterosexuals (Scandurra et al., 2021).

### Limitations and Suggestions for Future Research

The findings of this study should be interpreted in light of several limitations. First, the survey did not include attention-check items. Although all responses were screened for inconsistencies, the absence of formal attention checks means that inattentive or mischievous responding cannot be entirely ruled out. Future research should incorporate such measures to enhance data quality and validity.

Second, the sampling method employed was non-probabilistic, which inherently limits the external validity of the research design and its findings. Nevertheless, this limitation is somewhat mitigated by the heterogeneous nature of the sample, which included individuals with diverse demographic characteristics. This was apparent in the multigroup analysis. The findings of variance at multiple levels (factor loadings, intercepts, and residuals) indicate that the HABS does not function equivalently across the two populations. This suggests that group comparisons using the HABS should be interpreted with caution, as observed differences may be due to measurement bias rather than true differences in heteronormative attitudes. Moreover, given the professional training that mental health practitioners receive in diversity and inclusion, their responses to heteronormativity-related items are likely to reflect their knowledge and exposure to contemporary gender and sexual diversity concepts. This divergence from the non-professionals population in interpreting heteronormative beliefs underscores the need for culturally sensitive adaptations of scales when applied across professional and non-professional groups (Chen, 2007).

Third, reliability and validity checks of the Greek version of the HABS were conducted collectively for all participants of each sample, as done by the developer of the HABS, and in the subsequent validation checks (e.g., Italy). However, the literature consistently shows that heterosexual individuals tend to exhibit higher levels of heteronormativity and homonegativity than LGBTQI+ individuals. Therefore, subsequent research examining the psychometric properties of the HABS could produce meaningful findings through renewed multigroup analyses across participants’ gender identities and sexual orientations.

Fourth, evidence for the discriminant validity of the Greek version of the HABS was limited to the negative correlations between its subscales and the personality trait Openness to experience. While this finding is consistent with prior literature suggesting that lower openness fosters rigid adherence to conventional gender and sexual norms, it underscores the need to test discriminant validity against a broader set of constructs beyond personality traits (e.g., general cognitive abilities or unrelated attitudes). Similarly, although convergent validity with religiosity was supported, this conclusion is constrained by the use of two ad hoc items to assess religiosity, which showed modest reliability (Spearman–Brown coefficient = .49). This limited measurement precision warrants caution when interpreting the strength of these associations. Future studies should therefore employ well-validated multidimensional instruments of religiosity and include additional theoretically relevant constructs (e.g., political ideology, authoritarianism, or right-wing authoritarianism) to provide a more robust and comprehensive test of both convergent and discriminant validity.

### Conclusion

Despite its limitations, the HABS appears to be a reliable and valid instrument that can meaningfully advance research on heteronormativity in Greece. It provides a valuable framework for expanding studies on heteronormativity and its substantial implications in the lives of individuals, particularly among women and LGBTQI+ populations. The present study confirmed the scale’s structural validity -both regarding the number and content of its dimensions- across general and specialized samples. Furthermore, the correlations with other measurements were consistent with findings from previous research (Habarth, 2015; Scandurra et al., 2021).

However, the observed variation in factor loadings, intercepts, and residuals between the two groups suggests that the HABS may not assess heteronormativity equivalently across populations. While the basic structure of the scale was supported, these discrepancies highlight the need for group-specific interpretations of heteronormative beliefs, especially when making comparisons between populations with varying degrees of exposure to concepts of gender and sexual diversity. In our sample, both mental health and non-mental health professionals scored below the theoretical median of the HABS, suggesting that heteronormativity and related negative beliefs may be less pervasive than expected and that Greek society may be gradually becoming more inclusive, although the significant differences observed between the groups imply that non-mental health professionals may not be advancing toward inclusivity at the same rate as their mental health counterparts. Future research should, however, aim to refine the HABS by developing alternative versions or adapting the existing scale for use among mental health professionals, thereby improving its applicability and validity in both professional and general populations.

The validation of the HABS holds important implications for both research and practice. In Greece, as in many other countries, there remains a pressing need for interventions, policies and legislation to be grounded in robust empirical evidence regarding attitudes towards sex and gender. The HABS can serve as a valuable tool in this context, informing targeted interventions, supporting educational initiatives and guiding evidence policy development.

## Data Availability

Data are available at: https://doi.org/10.17605/OSF.IO/678AE

## Footnotes

1 The Kuder-Richardson Formula 20 (KR-20) is a coefficient of internal consistency used for measures with dichotomous items (e.g., 0/1 responses), analogous to Cronbach’s alpha for binary data.

## Code Availability

Non applicable.
